# Patient Preferences for the Management of Gastrointestinal Symptoms in Kidney Transplantation: a Discrete Choice Experiment

**DOI:** 10.1016/j.ekir.2023.07.034

**Published:** 2023-08-12

**Authors:** Tess E. Cooper, Amy Dalton, Anh Kieu, Ryan Gately, Michael J. Bourke, Jonathan C. Craig, Rabia Khalid, Wai H. Lim, Nicole Scholes-Robertson, Armando Teixeira-Pinto, Allison Jaure, Germaine Wong, Martin Howell

**Affiliations:** 1 Cochrane Kidney and Transplant, The Children's Hospital at Westmead, Australia; 2 Sydney School of Public Health, The University of Sydney, Australia; 3 Center for Kidney Research, The Children’s Hospital at Westmead, Australia; 4 Princess Alexandra Hospital, Australia; 5 School of Medicine, The University of Queensland, Australia; 6 Westmead Hospital, Australia; 7 Sydney Medical School, The University of Sydney, Australia; 8 College of Medicine and Public Health, Flinders University, Australia; 9 Sir Charles Gairdner Hospital, Australia; 10 School of Medicine, University of Western Australia, Australia

**Keywords:** chronic kidney disease, discrete choice experiment, gastrointestinal symptoms, kidney transplant

## Abstract

**Introduction:**

Gastrointestinal (GI) symptoms in kidney transplant are common and debilitating. We aimed to ascertain patients’ preferences for GI symptom management options to help future interventions align with treatment priorities.

**Methods:**

A discrete choice experiment was conducted with kidney transplant recipients in 3 Australian nephrology units. A multinomial logit model was used to quantify the preferences and trade-offs between 5 characteristics: cost, formulation, symptom burden, dietary changes, and medication quantities.

**Results:**

Seventy patients participated (mean age ± SD: 47 ± 15 years, 56% female), 57% had GI symptoms. Patients preferred interventions that will achieve complete resolution of GI symptoms compared to no improvement (odds ratio [95% confidence interval]: 15.3 [1.80, 129.50]), were delivered as a tablet rather than a sachet (1.6 [1.27, 2.08]), retained their current diet compared to eliminating food groups (6.0 [2.19, 16.27]), reduced medication burden (1.4 [1.06, 1.79]), and had lower costs (0.98 [0.96, 1.00]). Participants would be willing to pay AUD$142.20 [$83.90, $200.40] monthly to achieve complete resolution of GI symptoms or AUD$100.90 [$9.60, $192.10] to have moderate improvement in symptoms.

**Conclusions:**

Interventions that are highly effective in relieving all GI symptoms without the need for substantive dietary changes, and in tablet form, are most preferred by kidney transplant recipients.

## Graphical abstract

**Figure:**
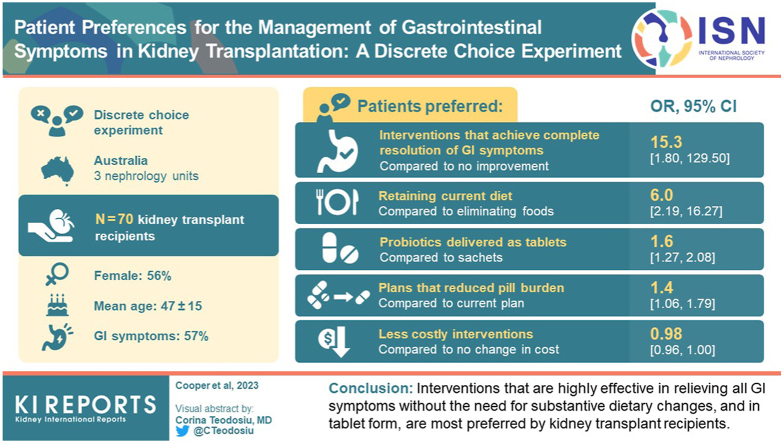

GI symptoms are frequently reported in patients with kidney and simultaneous pancreas-kidney (SPK) transplant recipients. Approximately 50% of patients experience some form of GI symptoms within the first year of transplantation.^1^ The symptom cluster includes diarrhea, constipation, bloating, abdominal pain, heart burn, and gastroesophageal reflux. These symptoms can be debilitating, impair quality of life, incur substantial morbidity burden, increase health care resource utilization, and are associated with allograft dysfunction.^2^

GI symptoms are usually due to the direct or indirect adverse effects of immunosuppressive medications, such as mycophenolate mofetil, mammalian target or rapamycin inhibitors, prednisolone, or tacrolimus.^2^ The most common approaches to management are to reduce the immunosuppressant dose or change the class of agent used. However, these changes may predispose recipients to higher risks of acute rejection.^1^^,^^3^, ^4^, ^5^ Apart from drug-related complications, other causes of GI symptoms may include opportunistic infections (such as cytomegalovirus and fungal infections), gastroparesis, poor quality diet, inflammatory bowel disease, gastrointestinal reflux disease, and occasionally, potentially serious reasons including malignancies.^2^^,^^3^

Distinguishing between GI symptoms that are caused by infections, drug therapy, or malignancy is often difficult but important to guide and inform treatment options. Interventions to address or reduce GI symptoms are multiple, and may include dietary changes, pharmacotherapy, or disease modifying agents such as repopulation of the GI system with normal intestinal microflora, using prebiotics, probiotics, or synbiotics.^1^^,^^6^^,^^7^ These management strategies have various characteristics, including costs, effectiveness, and side effects profile, that may directly influence clinicians’ and patients’ choices for one option over the other. Currently, decisions for the management of these symptoms for transplant recipients are predominantly made by clinicians, who may not have the preferences, priorities, and treatment goals as patients.^7^ Considering that GI disturbances remain a substantial burden for patients with kidney and SPK transplants, a better understanding of patients’ preferences in relation to the management characteristics may inform the design and development of novel approaches to address this patient-important and relevant outcome. Incorporating patient’s perspectives in clinical decision-making about the choice of interventions that best align with their needs will also facilitate optimization of the allocation of scarce resources.

### Objective

This study aimed to elicit patients’ preferences regarding the management of gastrointestinal symptoms following kidney and SPK transplantation.

## Methods

This study is registered on the Australian New Zealand Clinical Trials Registry: ACTRN12621000548831, published in our protocol,^8^ and has been approved by the Western Sydney Local Health District Human Research Ethics Committee of New South Wales Health (HREC ETH03007). This study is developed and reported in accordance with the reporting checklist for observational studies (STROBE)^9^ (Supplementary Table S1).

### Setting and Recruitment

Participants were recruited from 3 nephrology units within The Western Renal Service (Western Sydney Local Health District and Nepean Blue Mountains Local Health District), New South Wales, Australia. Recruitment was conducted from January 2021 to June 2021. All eligible patients were identified using the renal databases and through communication with medical and nursing staff.

### Participants

#### Eligibility Criteria

Adults 18 years and older, kidney or SPK transplant recipients with 1 functioning kidney and/or pancreas allograft, were eligible to participate. Participants were included irrespective of whether they had current or past experiences of GI symptoms.

#### Exclusion Criteria

Patients with prior bowel resection, chronic pancreatic insufficiency, recent infection-related diarrhea; transplant recipients who have had a recent rejection episode in the last 3 months; or patients who were unable to provide informed consent were excluded from the study.

### Discrete Choice Experiment Methodology

We administered a discrete choice experiment (DCE) survey to quantify patient preferences of management strategies (attributes) of GI symptoms in kidney transplant recipients. A DCE aims to understand the preferences and trade-offs that individual patients are willing to make between different aspects of their healthcare and their own health behaviors.^10^ A DCE design is an attribute-based approach to eliciting stated-preferences. Participants are presented with hypothetical scenarios (choice sets) composed of 2 or more alternate attributes.^11^ Along with the “discrete” feature, the DCE design forms the generation and analysis of choice data in order to identify the value that an individual places on the given attributes when weighing up multiple or complex treatment options.^11^^,^^12^ Discrete choice experiments are widely used in health care due to their ability to provide the opportunity to elicit an answers from patients across a range of research questions (some of which cannot otherwise be satisfactorily answered as isolated questions) to identify an acceptable comprehensive treatment plan when treatment considerations are multifactorial.^13^ These circumstances are common in chronic diseases often accompanied by comorbidities, such as chronic kidney disease and solid organ transplantation.

### DCE Survey Design

The survey was developed following a review of the literature, in-depth collaboration with consumer partners, discussion among the research team (consisting of clinicians and researchers), and preliminary qualitative interviews (semistructured in-depth qualitative interviews^8^) with patients and consumers to ensure the inclusion of a broad range of relevant, clinically important and patient-important outcomes, symptoms and management characteristics of cost, formulation, diet changes, GI symptoms, and medications.^14^, ^15^, ^16^, ^17^, ^18^ After the survey was developed, it was piloted with our consumer partners to ensure face and content validity. The intention of this survey was not to amend current clinical practice, but to discover complimentary management strategies to aid existing medical treatments that cause serious GI side effects. We used *a priori* expectations to set priors for the initial design and then piloted for face validity; however, we did not change the design based on the pilot (including priors). In accordance with common practice,^19^^,^^20^ we elected not to include additional questions to check for reliability and consistency of the survey response in order to limit survey burden.

#### Attributes

**Figure 1:**
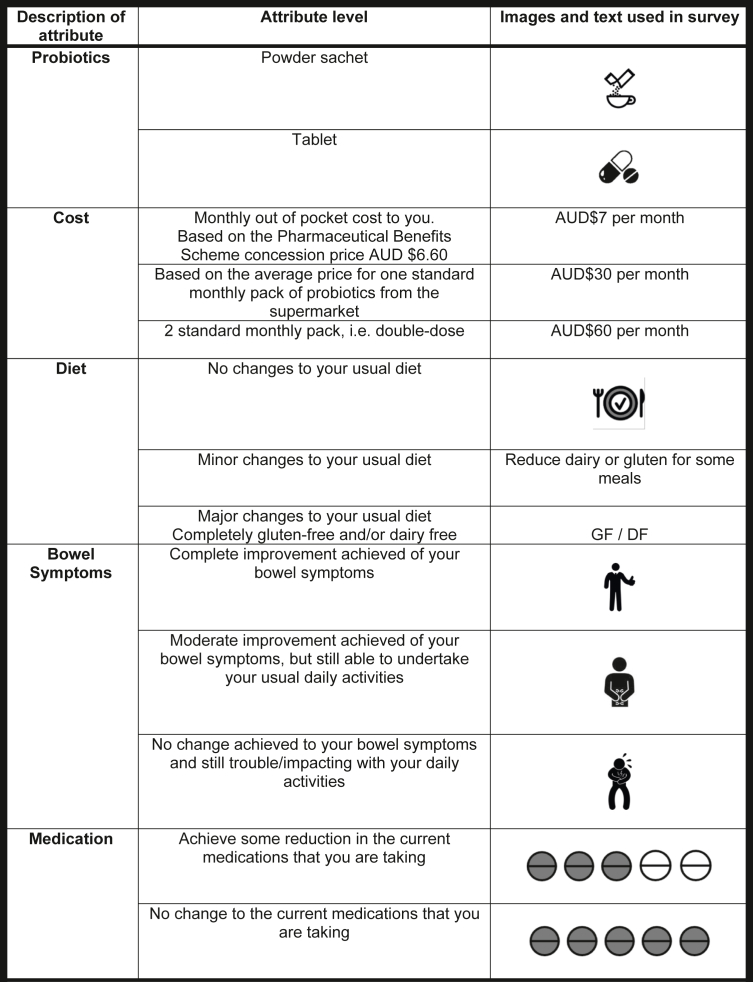
Choice sets and attribute level options. AUD$, Australian Dollars; DF, dairy free; GF, gluten free; PBS, Pharmaceutical Benefits Scheme, Australian government subsidizing scheme for medications.

The survey design (Figure 1) encompassed 5 attributes describing the characteristics of treatment for GI symptoms with 2 or 3 levels incorporated:

1. Formulation (probiotics as an example): (i) tablet and (ii) sachet powder.
2. Average cost to the patient per month: (i) AUD$7, (ii) AUD$30, and (iii) AUD$60.
3. Dietary changes to the patient’s usual diet: (i) no changes required to be made; (ii) minor changes such as reducing aggravating foods like dairy, gluten, sugars, alcohol, and fatty fast foods; and (iii) major changes such as eliminating dairy and/or gluten.
4. Changes to patient’s current bowel symptoms: (i) complete improvement of patient bowel symptoms is achieved; (ii) moderate improvement of bowel symptoms are achieved, but still able to undertake usual daily activities; (iii) No change in bowel symptoms is achieved and the patient still experiences troubles impacting daily activities.
5. Changes in currently prescribed medications: (i) able to achieve some reduction in the current medications the patient is taking for their transplant and (ii) no change able to be made to the current medications that the patient is taking.

For each level, the study personnel used the list above and provided to the patients a detailed verbal explanation of what these meant. Study personnel explained what each level entailed, differences, and that these are examples but not an exhaustive list. Study personnel ensured that participants understood what was being asked before they started the questions.

#### Justification for Attributes

Interventions to address or reduce GI symptoms are multiple, and may include dietary changes, pharmacotherapy, or disease modifying agents such as repopulation of the GI system with normal intestinal microflora, using prebiotics, probiotics, or synbiotics.^1^^,^^6^^,^^7^ Although there is limited emerging evidence to show efficacy of prebiotics or probiotics over other intervention treatments, probiotics were chosen as the hypothetical intervention in this DCE because it is a supplement that transplant recipients are familiar with, can be purchased over-the-counter, and thus far has limited reported side effects.^21^^,^^22^ In addition to the practicalities of probiotics, when taken in certain quantities, probiotics have been shown to safely restore altered intestinal microbiota and alleviate GI symptoms in patients with chronic kidney disease to a tolerable level.^21^^,^^22^ Formulation was chosen as an attribute because the preference for tablets or powder within this specific population is necessary to ensure uptake. Cost was based on either (i) the Australian Pharmaceutical Benefits Scheme co-payment of AUD$6.80^23^ for concession card holders rounded to $7 for ease of interpretation; or (ii and iii) based on the average retail cost of 1 packet of 30 probiotics tablets that can be purchased over-the-counter in Australia, AUD$30 for 1 packet, or AUD$60 based on 2 packets (i.e., a double dose). Dietary change was chosen as an attribute because multiple interventions are often required to address GI issues, and making changes to daily foods is often prescribed hand-in-hand with nutritional supplements such as probiotics to assist changes to the intestinal microflora balance.^6^^,^^7^ Changes to patients’ bowel symptoms and changes to currently prescribed medications were chosen as attributes based upon our preliminary qualitative work and in-depth discussions with our patient partners. Both attributes were highlighted as desired outcomes of GI issues in the kidney population due to the already cumbersome mental and physical load that their chronic disease encompasses.

Of the 5 attributes and 12 levels, there were 792 possible combinations; therefore, a Bayesian D-efficient design was used with estimated priors using the Ngene software^24^ to come up with a practical number for completion of 25 choice sets each containing 2 unlabeled treatment options, grouped into 5 blocks each with 5 choice sets.^25^ A second aim for using a blocked design was to have a simple survey that could be completed within 5 to 10 minutes, potentially within the clinical setting, or at home.

The utility function used was: Utility = β_cost_∗COST + β_admsatchet_∗ADMSATCHET + β_admtablet_∗ADMTABLET + β_dietminorchange_∗DIETMINORCHANGE + β_dietmajorchange_∗DIETMAJORCHANGE + β_symptnoimprove_∗SYMPTNOIMPROVE + β_symptmodimprove_∗SYMPTMODIMPROVE + β_symptcompleteimprove_∗SYMPTCOMPLETEIMPROVE + β_medsomereduct_∗MEDSOMEREDUCT + β_mednoreduct_∗MEDNOREDUCT.

**Figure 2:**
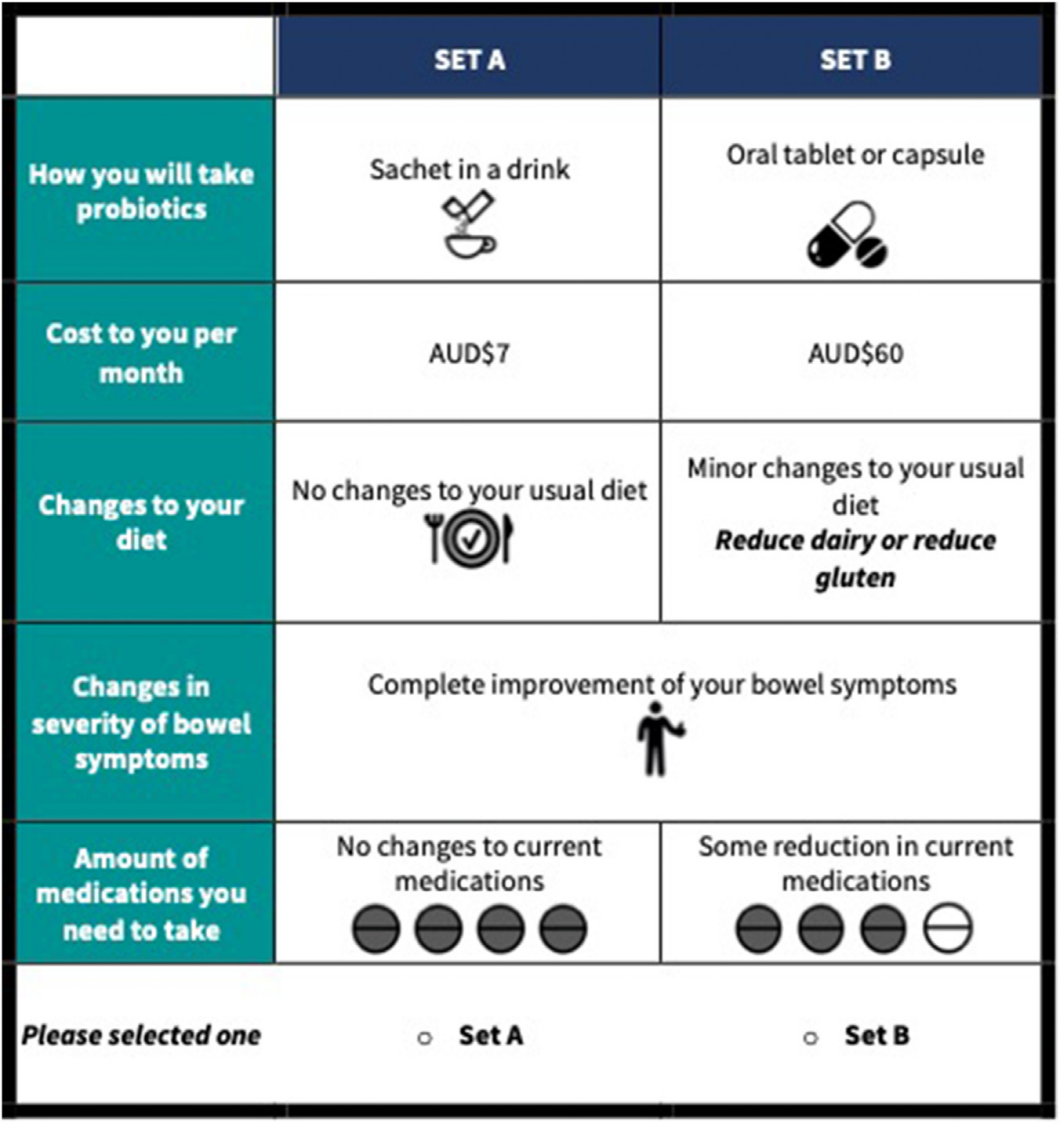
Example of discrete choice experiment questions within survey. AUD$, Australian Dollars.

The survey was framed to elicit relative preferences or importance of attributes of treatment regimen and outcomes rather than whether participants would accept one option over another or, none.^26^ Therefore, the DCE did not include an opt-out or neither choice. The survey was administered in-person using a tablet linked to the Qualtrics survey platform and participants were randomly allocated to 1 of the 5 blocks. An example of a single choice tasks is shown in Figure 2.

### Data Collection

Following consent, baseline demographic and medical characteristics were recorded via a paper questionnaire with the assistance of study personnel where needed. Participants then privately completed the full electronic survey directly after attending their usual medical appointment using a tablet provided by the study (Supplementary Figure S1).

### Sample Size

A Bayesian D-efficient design was used with estimated priors using the NGENE software.^24^ Based on the S-error term for the efficient design a sample size of 60 to 70 was expected to provide statistically robust parameter estimates.

### Statistical Analyses

We assessed the influence of the baseline demographic characteristics of each participant using stratification. To further assess preference heterogeneity, we also assessed the sociodemographic factors by inclusion as interaction variables within the regression model and with latent class modeling. We used NLOGIT version 6.0^27^ to analyze the choice set data. Analyses were conducted using a multinomial logit model assuming a linear additive utility function. Given that the DCE was unlabeled, constants were not included. The statistical significance of a coefficient (*P* < 0.05) signified the attribute as important to participants in determining their preferences. Because the DCE was unlabeled, constants are not considered relevant for the final model; however, in order to check for possible left to right bias, we conducted an analysis with a constant. Attribute levels were dummy coded except for costs which was included as a continuous variable. Validity was assessed by the extent to which results were consistent with the researchers’ prior expectations, with internal validity assessed by examining the signs and significance of parameter estimates.^12^ For ease of interpretation, the coefficients were expressed as odds ratios. The willingness to pay was calculated by the marginal rate of substitution method reported in Australian Dollars and US Dollars for international applicability. Privacy, storage, and data retention methods are described in our a priori published protocol.^8^

## Results

### Characteristics of Participants

Of the 72 eligible participants invited to participate in the study, 70 completed the survey, 1 did not respond, and 1 declined because they were not interested (Supplementary Figure S2). The mean age ± SD of the cohort was 47 ± 15 years, with 56% being female, all English-speaking, and 63% had a body mass index of less than 30 kg/m^2^. All participants had at least 1 comorbidity as follows (Table 1): hypertension (66%), diabetes (27%), dyslipidemia (23%), cardiovascular disease (19%), stroke (6%). All participants had at least one gastrointestinal-related symptom as follows (Table 1): gastroesophageal reflux disease (49%), other GI (bloating/distended stomach, bowel obstruction, chronic constipation, diarrhea, feeling fullness, gastroparesis, gut pain, nausea, urgency, or vomiting) (33%), irritable bowel disease (14%), or celiac disease (1%). Sixty-seven percent of participants had never smoked. The median time since their last transplant was 20 months (interquartile range 1.75 to 77). Twenty-three percent of participants had an SPK transplant and 74% received their organ from a deceased donor. All patients were taking immunosuppressive medications at the time of the study as follows (Supplementary Table S2): most commonly prednisolone (100%), tacrolimus (90%), and mycophenolate acid (76%). Other most common categories of medications taken regularly by participants were antireflux medications (80%) and antibiotics (80%). There was no missing data for any of the variables collected.

**Table 1:** Participant demographics and characteristics (*N* = 70)

Characteristic	*n* (%)
Age (yrs)	
18–39	25 (36)
40–59	26 (37)
>60	19 (27)
Sex	
Female	39 (56)
Male	31 (44)
BMI kg/m^2^	
< 18.5 (underweight)	2 (3)
= 18.5–24.9 (healthy)	21 (30)
= 25.0–29.9 (overweight)	21 (30)
> 30 (obese)	26 (37)
Current marital status	
Single or partnered	28 (54)
Married or de facto	36 (51)
Separated, divorce, or widowed	6 (9)
Education	
< year 12	16 (23)
HSC or equivalent to year 12	13 (19)
TAFE diploma or certificate	20 (29)
University degree	21 (30)
Current employment situation	
Working full-time or part-time	26 (37)
Student	3 (4)
Retired (for age or medical reasons)	18 (26)
Not currently working (any reason)	23 (33)
Ethic background	
Aboriginal or Torres Strait Islander	2 (3)
Anglo-Caucasian	31 (44)
Asian	16 (23)
Middle Eastern	7 (10)
Pacific Islands	4 (6)
Other European	5 (7)
Other^a^	5 (7)
Comorbidities	
Diabetes	19 (27)
Cardiovascular disease	13 (19)
Hypertension	44 (63)
Stroke	4 (6)
Dyslipidaemia	16 (23)
Gastrointestinal-related conditions and symptoms	
Coeliac disease	1 (1)
GERD or reflux symptoms	34 (49)
Irritable bowel syndrome	10 (14)
Other^b^	23 (33)
Smoking	
Never smoked	47 (67)
Current smoker	1 (1)
Ex-smoker	22 (31)
Time since last transplant (mo)	20 (median); 1.75–77 (IQR)
Transplant type	
Kidney only	54 (77)
Simultaneous pancreas-kidney	16 (23)
Donor type	
Living donor	18 (26)
Deceased donor	52 (74)

BMI kg/m^2^, body mass index in kilograms per meter squared; GERD, gastroesophageal reflux disease; HSC, higher school certificate; IQR, interquartile range; TAFE, technical and further education; <, less than; >, greater than.

a Other ethnicities: African American (1), Hispanic (1), Indian (1), Turkish (2).

b Other GI symptoms: bloating/distended stomach, bowel obstruction, chronic constipation, diarrhea, feeling fullness, gastroparesis, gut pain, nausea, ulcerative colitis, urgency, vomiting.

### Preferred Management Plan of GI Symptoms

**Figure 3:**
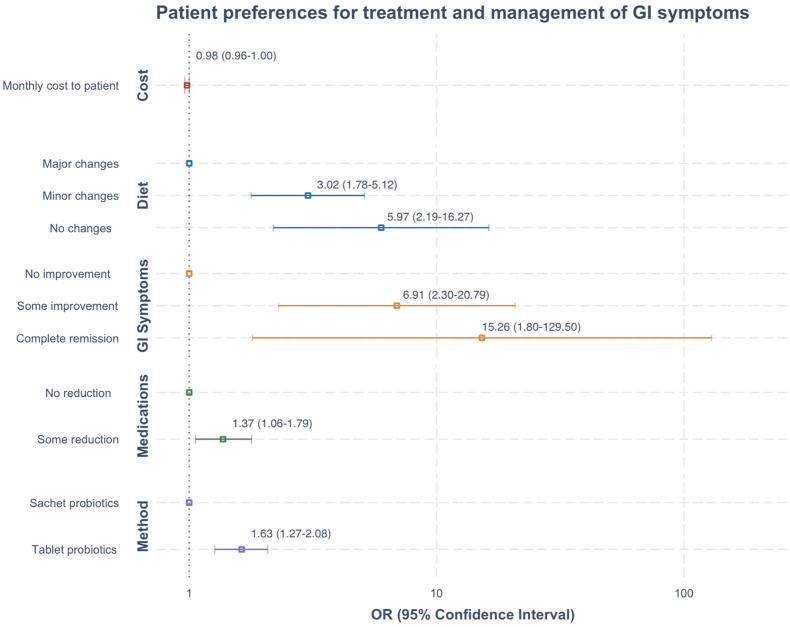
Patient preferences for treatment and management of GI symptoms. GI, gastrointestinal; OR, odds ratio.

Findings from the multinomial logistic regression analyses are summarized in Table 2 (model output is included in Supplementary Table S3). The model shows that all 5 attributes are relevant and contributed to the choices and in the expected direction (*P* < 0.05). For ease of interpretation, the coefficients are expressed as odds ratios (Table 2 and Figure 3). Of the 5 attributes, the most important for patients was changes in GI symptoms. Participants preferred a management plan that could achieve complete resolution of GI symptoms (odds ratio [95% confidence interval]: 15.3 [1.80–129.50]) or provided some improvement of GI symptoms (6.91 [2.30–20.79]), compared to no improvement in GI symptoms. The next most important attribute to patients was changes required to their usual diet. Transplant recipients preferred a management plan that would avoid substantial modifications to their current diet (5.97 [2.19–16.27]), followed closely by a plan that only required minor changes to their usual diet (reducing aggravating foods like dairy, gluten, sugars, alcohol, and fatty fast foods) (3.02 [1.78–5.12]), when compared to a management plan that required major dietary changes (such as eliminating dairy and/or gluten) (Table 2). Of the remaining 3 attributes, patients preferred to take the intervention in tablet form compared to sachets (1.6 [1.27–2.08]), a plan that would reduce their pill burden compared to current management (1.4 [1.06–1.79]), and less costly interventions (0.98 [0.96–1.00]).

**Table 2:** Patient preferences for GI treatment options

Attribute and Level	Coefficient	Odds ratio^a^ (95% CI)	*P*-value	WTP AUD$ (95% CI)	WTP USD$ (95% CI)
Monthly cost to the patient	−0.02	0.98 (0.96–1.00)	0.03	N/A	N/A
Formulation					
Tablet probiotics	0.49	1.63 (1.27–2.08)	< 0.001	$25.34 ($-30.59–$81.27)	$16.44 ($-19.85–$52.74)
Sachet probiotics	Reference	1		$0	$0
Changes required to usual diet					
No changes	1.79	5.97 (2.19–16.27)	< 0.001	$93.21 ($14.59–$171.83)	$60.49 ($9.47–$111.51)
Minor changes	1.11	3.02 (1.78–5.12)	< 0.001	$57.66 ($-7.29–$122.62)	$37.42 ($-4.73–$79.58)
Major changes	Reference	1		$0	$0
Changes in bowel symptoms					
Complete resolution	2.73	15.26 (1.80–129.50)	0.01	$142.15 ($83.89–$200.41)	$92.25 ($54.44–$130.06)
Some improvements	1.93	6.91 (2.30–20.79)	< 0.001	$100.86 ($9.58–$192.14)	$65.45 ($6.22–$124.69)
No improvement	Reference	1		$0	$0
Changes to current medications					
Some reduction	0.32	1.37 (1.06–1.79)	0.02	$16.53 ($-25.70–$58.76)	$10.73 ($16.68–$38.13)
No changes	Reference	1		$0	$0

AUD$, Australian Dollars per month; CI, confidence interval; N/A, not calculated for monthly cost to the patients; *P*: *P*-value of significance at 5%; SE, standard error; USD$, US Dollars based on exchange rate in September 2022; WTP, willingness to pay.

a The odds ratio is the exponential of the coefficient.

In the subgroup analyses of these patient preferences, the relative impact on these attributes did not differ significantly by sex, age group, or time elapsed since transplant (Table 3; Supplementary Table S4). Similarly, assessment of preference heterogeneity by latent class analysis did not identify any significant classes with differing groupings of preferences.

**Table 3:** Patient preference of GI treatment options – by subgroups of selected demographic characteristics

	Odds ratio (95% confidence interval)						
	Monthly cost to the patient	Formulation of taking probiotics	Changes required to diet		Changes in bowel symptoms		Changes to medications
		Tablet vs. sachet^a^	No changes vs. major changes^a^	Minor changes vs. major changes^a^	Complete resolution vs. no improvement^a^	Some improvement vs. no improvement^a^	Some reduction vs. no changes^a^
All	0.98 (0.96–1.00)	1.6 (1.27–2.08)	6.0 (2.19–16.27)	3.0 (1.78–5.12)	15.3 (1.80–129.50)	6.9 (2.30–20.79)	1.4 (1.06–1.79)
Sex							
Female	0.97 (0.95–1.00)	1.5 (1.02–2.14)	10.2 (2.29–44.89)	4.3 (1.93–9.65)	72.3 (2.89–1806.98)	12.6 (2.52–62.68)	1.3 (0.84–1.84)
Male	0.98 (0.96–1.01)	1.8 (1.29–2.54)	4.5 (1.09–18.58)	2.5 (1.20–5.01)	5.5 (3.63–110.92)	5.1 (1.05–25.04)	1.5 (1.05–2.21)
Age							
18–39 yr	0.98 (0.95–1.01)	1.4 (0.96–2.17)	3.6 (0.72–18.12)	2.1 (0.87–5.17)	8.6 (0.23–327.19)	8.0 (1.21–52.26)	1.3 (0.87–2.06)
40–59 yr	0.99 (0.96–1.02)	1.4 (0.89–2.05)	8.3 (1.39–49.83)	4.0 (1.66–9.67)	16.4 (0.32–851.30)	7.8 (1.11–54.68)	1.4 (0.86–2.22)
60+ yr	0.97 (0.93–1.00)	2.7 (1.57–4.51)	11.9 (1.28–111.54)	4.6 (1.33–15.80)	54.4 (0.68–4373.46)	5.5 (0.52–59.33)	1.5 (0.84–2.73)
Time since Tx							
≥100 wk	0.99 (0.97–1.02)	1.4 (0.94–1.95)	5.0 (1.13–22.40)	3.0 (1.35–6.45)	3.6 (0.15–88.84)	5.4 (1.04–27.88)	1.3 (0.83–1.87)
<100 wk	0.97 (0.95–1.00)	1.9 (1.32–2.63)	7.3 (1.77–29.69)	3.2 (1.54–6.69)	50.4 (2.43–1044.52)	8.1 (1.73–38.13)	1.4 (1.00–2.04)

AUD$, Australian Dollars; CI, confidence interval; *P*, *P*-value of significance at 5%; SE, standard error; Tx, transplant; wk, weeks; yr, years.

a Reference level.

### Willingness to Pay AUD$ Per Month

Compared to achieving no improvement in symptoms, the willingness to pay per month to achieve complete resolution of GI symptoms or to achieve moderate improvement in GI symptoms was (AUD$ [95% confidence interval]): $142.2 [$83.9–$200.4] and $100.9 [$9.6–$192.1], respectively (see Table 2 for US Dollars). Compared to making major changes to their current diets, participants were willing to pay $93.21 [$14.59–$171.83] per month to avoid making any changes to their current diets and $57.66 [$-7.29–$122.62] per month to only make minor changes to their diets. Participants were willing to pay $25.34 [$-30.59–$81.27] per month to be able to take probiotics in tablet form instead of sachets.

## Discussion

Findings from this study suggested that alleviation of the severity of GI symptoms, minimizing dietary changes, and formulation all influenced patients’ preferences in the management of GI symptoms after transplantation. Kidney and SPK transplant recipients preferred interventions that provided complete resolution of symptoms over plans that achieved moderate or no improvement in symptoms. There was also a preference to making no change or minimal changes to their current diet compared to elimination of certain foods such as highly processed foods, high sugar foods, high fatty foods, dairy, and gluten. Taking the intervention in tablet form was the preferred treatment modality over sachets. Of less importance was reducing pill burden and the costs of the treatment. The high priority of GI symptom control is indicated by the high estimated willingness to pay (up to AUD$142.50 per month) to be symptom free. The subgroup and latent class analyses showed that these strong preferences for management of GI symptoms did not differ significantly by sex, age group, or time elapsed since transplant. The keen desire to be symptom free suggested a management plan that provided the greatest relief from GI symptoms was most preferred by patients, irrespective of the patients’ characteristics and duration since the transplant surgery.

It is well known that GI symptoms are highly prevalent in transplant recipients and significantly affect their quality of life.^7^^,^^28^, ^29^, ^30^ Prior research has identified GI symptoms as the third most common complication in solid organ transplant recipients.^31^^,^^32^ These adverse effects including diarrhea, constipation, severe reflux, and fullness are frequently associated with immunosuppression and other posttransplant medications, including prophylactic antibiotics and antifungal treatments. These symptoms can have serious consequences and influences on the daily living, quality of life, and life participation of transplant recipients. Despite these symptoms being common, there are limited intervention studies addressing similar conditions or strategies and they are also known to be poorly addressed by clinicians. Prior studies, however, have not quantified the values of the attributes that defined management regimens for GI symptoms. Our study is novel because it addresses a common and enduring concern for transplant recipients. It has also identified the key features and their relative importance using a “willingness to pay” approach. These findings can be used to inform the design and development of patient-centered interventions in future trials.

The Standardized Outcomes in Nephrology initiative has developed the core outcomes for kidney transplantation trials^33^ that show a strong focus on allograft health (including kidney function and allograft loss), serious adverse outcomes of cardiovascular disease, cancer and infection, mortality, and life participation. Although not a core outcome, GI disorders were identified as important outcomes by patients, caregivers, and health professionals. Furthermore, GI symptoms are substantial contributors to reduced life participation. The clinical relevance of this study is that we have identified how kidney transplant recipients would prefer to receive or take complimentary management strategies for their severe GI symptoms and the desired outcomes. The results suggest that patients would take probiotics (even at a relatively high cost) in order to reduce, or completely resolve, their GI symptoms. This is an important finding for clinicians when discussing treatment and management options for kidney transplant recipients who report severe GI symptoms. Currently, where GI symptoms are assessed as being due to adverse effects of the immunosuppressive medications,^2^^,^^3^ changing the medication or lowering the dose of the immunosuppression may alleviate GI symptoms but may also increase the risk of graft rejection.^1^^,^^3^, ^4^, ^5^, ^6^ Reducing immunosuppressive medications to treat or address GI symptoms may not be reflective of patients’ preferences given a strong and often overwhelming focus on graft function and survival.^31^ This study has identified preferences for a complimentary strategy or intervention (such as probiotics) that addresses the debilitating effects of GI symptoms. However, these types of interventions have yet to be shown to achieve the outcomes identified in this study (i.e., major reduction in symptoms with minimal change to diet).^34^ In the context of shared decision making, it is important to communicate the balance of benefits and harms recognizing patients’ preferences which, until effective complimentary interventions are available, will likely be a balance between graft function and tolerating GI symptoms.

Our study highlights the critical importance of better understanding of patient preferences between health care providers and transplant recipients to address the challenging and unmet needs of GI management. Common themes reported by prior studies indicated that the embarrassment, inconvenience, and difficulties in dealing with the GI symptoms are unbearable for some patients.^35^ Treatment options are often limited, with many untested, and unproven agents being promoted and sold widely over-the-counter as nonprescription therapeutics. A shared decision-making process is needed to ensure that health care professionals engage patients and their families to make informed decisions about patients’ own health. This requires a 2-way discussion about the risks and benefits of the available management options, taking into consideration the values, preferences, and the circumstances of the individuals. Knowledge of potential barriers, such as the affordability of treatment options and hurdles to implement, can inform clinicians and patients about the management choices and policy planning stratified based on the individual’s symptoms and disease severity.

### Strengths and Limitations

Our study has several strengths. Using a well-established and validated method to elicit and quantify treatment preferences of GI symptoms and disorders, findings from this study are novel and important additions to the existing literature described by the impact of adverse side effects and quality of life in kidney transplantation.^7^^,^^28^, ^29^, ^30^ We performed subgroup and latent class analyses, which supported a high degree of homogeneity in the preferences across patients with differing demographic and clinical characteristics.

This study, however, has some potential limitations. The study findings may not be generalizable to other transplant settings because the participants were recruited from 3 nephrology units within Western Sydney, Australia. Second, this DCE elicited stated preferences for choices based on hypothetical scenarios and not the actual choices made by the individuals. The participants chose the alternatives (treatment options) that best reflected their values and preferences, and estimates will to some extent, be influenced by the elicitation framework and scenarios. Third, the willingness to pay is based on hypothetical scenarios presented and reflect the relative importance of the attributes; however, the DCE did not include a ‘neither’ option which can lead to an overestimation of the willingness to pay. Fourth, GI symptoms cannot be considered in isolation. In this study, we have assessed and measured the preferences of transplant recipients; however, many of these GI disturbances may have already been developed during their predialysis and dialysis stages. Fifth, a further drawback of the DCEs is that some other important attributes might have been missed, even where a robust process has been followed.^11^^,^^12^ This is possible for our study; however because it is not possible to include all possible attributes in a DCE, following usual practice, the included attributes were based on current evidence within the literature and using an iterative consumer-clinician-researcher process. Sixth, the pilot testing was undertaken to inform face validity; however, the possible access to sample size in the patient population at the clinic was not sufficient for the pilot to assess validity of the priors used in the Bayesian sample design. Further to this, the estimate of sample size was based on a main effects model and not a latent class model. A larger sample size may have identified statistically significant latent classes.

In conclusion, this study has quantified and established the relative importance of the various attributes for managing GI symptoms, and the extent to which the participants are willing to trade between these characteristics in kidney and SPK transplant recipients. Strategies that relieve and resolve all GI symptoms without the need for substantive dietary changes and modifications are most preferred by transplant recipients.

## Disclosure

All the authors declared no competing interests.

## Supplementary Materials

Supplementary File (PDF)

**Figure S1.** Full electronic survey.

**Figure S2.** Study design and cohort flow chart of participation.

**Table S1.** STROBE Statement.

**Table S2.** Participant current medications.

**Table S3.** Model output with and without a constant.

**Table S4.** Participant demographics stratified by randomized blocks 1 to 5.
