# Supplementary material for: Patient Preferences for the Management of Gastrointestinal Symptoms in Kidney Transplantation: a Discrete Choice Experiment
Source: Kidney Int Rep. 2023 Aug 12;8(10):1978–88. doi: 10.1016/j.ekir.2023.07.034 (PMC10577360; doi:10.1016/j.ekir.2023.07.034)
Supplement: Supplementary File (PDF) [file mmc1.pdf]

SUPPLEMENTARY MATERIALS

LIST OF SUPPLEMENTARY MATERIALS

- **Table S1:** STROBE Statement
- **Table S2:** Participant current medications
- **Table S3:** Model output with and without a constant
- **Table S4:** Participant demographics stratified by randomized blocks 1 to 5
- **Figure S1:** Full electronic survey
- **Figure S2:** Study design and cohort flow chart of participation

Table S1: STROBE Statement checklist of items that should be included in reports of *cross-sectional studies*

von Elm E, Altman D, Egger M, Pocock S, Gotzsche P, Vandenbroucke J, and STROBE Initiative. The Strengthening the Reporting of Observational Studies in Epidemiology (STROBE) statement: guidelines for reporting observational studies. *Journal of Clinical Epidemiology*. 2008;61(4):344-349.

Vandenbroucke JP, von Elm E, Altman DG, Gøtzsche PC, Mulrow CD, Pocock SJ, Poole C, Schlesselman JJ, Egger M, for the STROBE Initiative. Strengthening the Reporting of Observational Studies in Epidemiology (STROBE): Explanation and Elaboration. *Epidemiology* 2007;18(6):805-835.

	**Item No**	**Recommendation**	**Page**
**Title and abstract**	1	(*a*) Indicate the study's design with a commonly used term in the title or the abstract	1,4
		(*b*) Provide in the abstract an informative and balanced summary of what was done and what was found	4
**Introduction**			
Background/rationale	2	Explain the scientific background and rationale for the investigation being reported	5,6
Objectives	3	State specific objectives, including any prespecified hypotheses	6
**Methods**			
Study design	4	Present key elements of study design early in the paper	6
Setting	5	Describe the setting, locations, and relevant dates, including periods of recruitment, exposure, follow-up, and data collection	6
Participants	6	(*a*) Give the eligibility criteria, and the sources and methods of selection of participants	7
Variables	7	Clearly define all outcomes, exposures, predictors, potential confounders, and effect modifiers. Give diagnostic criteria, if applicable	8,9,10
Data sources/ measurement	8*	For each variable of interest, give sources of data and details of methods of assessment (measurement). Describe comparability of assessment methods if there is more than one group	11
Bias	9	Describe any efforts to address potential sources of bias	8-13
Study size	10	Explain how the study size was arrived at	11
Quantitative variables	11	Explain how quantitative variables were handled in the analyses. If applicable, describe which groupings were chosen and why	8-13
Statistical methods	12	(*a*) Describe all statistical methods, including those used to control for confounding	11-12
		(*b*) Describe any methods used to examine subgroups and interactions	11-12
		(*c*) Explain how missing data were addressed	11-12
		(*d*) If applicable, describe analytical methods taking account of sampling strategy	11-12
		(*e*) Describe any sensitivity analyses	11-12
**Results**			
Participants	13*	(a) Report numbers of individuals at each stage of study - e.g., numbers potentially eligible, examined for eligibility,	12

		confirmed eligible, included in the study, completing follow-up, and analysed	
		(b) Give reasons for non-participation at each stage	12, Figure S2
		(c) Consider use of a flow diagram	Figure S2
Descriptive data	14*	(a) Give characteristics of study participants (e.g., demographic, clinical, social) and information on exposures and potential confounders	12-13, Table 1
		(b) Indicate number of participants with missing data for each variable of interest	13
Outcome data	15*	Report numbers of outcome events or summary measures	13-14 Table 1
Main results	16	(*a*) Give unadjusted estimates and, if applicable, confounder-adjusted estimates and their precision (e.g., 95% confidence interval). Make clear which confounders were adjusted for and why they were included	13-14
		(*b*) Report category boundaries when continuous variables were categorized	13-14
		(*c*) If relevant, consider translating estimates of relative risk into absolute risk for a meaningful time period	13-14
Other analyses	17	Report other analyses done - e.g., analyses of subgroups and interactions, and sensitivity analyses	Table 2, Table 3, Table S3
**Discussion**			
Key results	18	Summarise key results with reference to study objectives	14
Limitations	19	Discuss limitations of the study, taking into account sources of potential bias or imprecision. Discuss both direction and magnitude of any potential bias	16
Interpretation	20	Give a cautious overall interpretation of results considering objectives, limitations, multiplicity of analyses, results from similar studies, and other relevant evidence	14-16
Generalisability	21	Discuss the generalisability (external validity) of the study results	14-16
**Other information**			
Funding	22	Give the source of funding and the role of the funders for the present study and, if applicable, for the original study on which the present article is based	19

Table S2. Participant current medications (N = 70)

Medications	n (%)
**Immunosuppressants**	
Azathioprine	8 (11)
Cyclosporin	5 (7)
Everolimus	1 (1)
Leflunomide	4 (6)
Mycophenolate acid	53 (76)
Prednisolone	70 (100)
Sirolimus	1 (1)
Tacrolimus	63 (90)
**Antihypertensives**	
Unspecified	56 (80)
Anti-platelets	2 (3)
Anti-coagulants	33 (47)
Calcium channel blocker	31 (44)
Beta-blocker	30 (43)
Central acting	4 (6)
Angiotensin-converting enzyme inhibitors/Angiotensin receptor blockers	15 (21)
Vasodilator	6 (9)
**Lipids**	18 (26)
**Oral hypoglycaemics**	11 (16)
**Insulin**	7 (10)
**Vitamins**	
Fish oil	3 (4)
Vitamin D	31 (44)
Calcium	30 (43)
Vitamin and mineral combinations	15 (21)
Magnesium	23 (33)
Folate	2 (3)
D-mannose	1 (1)
Sodium bicarbonate	10 (14)
Probiotics	2 (3)
**Antiviral agents**	35 (50)
**Antibiotics**	56 (80)
**Antifungals**	19 (27)
**Diuretics**	8 (11)
**Laxatives**	10 (14)
**Reflux medications**	56 (80)
**Anti-diarrhoea medications**	1 (1)
**Antidepressants**	8 (11)
**Analgesics**	4 (5)

N: total sample size; **n:** number; **%:** percentage.

Table S3: Model output with and without a constant

i. Model without constant

Choice	Coefficient	Standard Error	Z	Prob. |z| >Z*	95% Confidence Interval	
**Cost**	-0.01917**	0.00908	-2.11	0.0348	-0.03697	-0.00137
**Administration of probiotics** Tablets	0.48579***	0.12497	3.89	0.0001	0.24085	0.73073
**Diet** No changes	1.78683***	0.51148	3.49	0.0005	0.78435	2.78931
**Diet** Minor changes	1.10541***	0.26946	4.10	0.0000	0.57728	1.63355
**Symptoms** Moderate improvement	1.93349***	0.56173	3.44	0.0006	0.83251	3.03447
**Symptoms** Complete improvement	2.72504**	1.09116	2.50	0.0125	0.58640	4.86369
**Medications** Some changes	0.31691**	0.13451	2.36	0.0185	0.05327	0.58055

Significant at 1%*; 5%**; 10%***
Log likelihood function -212.28540
Estimation based on N = 350, K = 7
Inf.Cr.AIC = 438.6
AIC/N = 1.253

ii. Model with constant

Choice	Coefficient	Standard Error	Z	Prob. |z| >Z*	95% Confidence Interval	
**Constant**	-0.02766	0.11820	-0.23	0.8150	-0.25933	0.20401
**Cost**	-0.01911**	0.00909	-2.10	0.0356	-0.03693	-0.00129
**Administration of probiotics** Tablets	0.48536***	0.12509	3.88	0.0001	0.24019	0.73053
**Diet** No changes	1.78601***	0.51180	3.49	0.0005	0.78290	2.78912
**Diet** Minor changes	1.10285***	0.26965	4.09	0.0000	0.57435	1.63135
**Symptoms** Moderate improvement	1.92866***	0.56176	3.43	0.0006	0.82763	3.02970
**Symptoms** Complete improvement	2.72673**	1.09179	2.50	0.0125	0.58686	4.86659
**Medications** Some changes	0.31325**	0.13502	2.32	0.0203	0.04862	0.57789

Significant at 1%*; 5%**; 10%***
Log likelihood function -212.25804
Estimation based on N = 350, K = 8
Inf.Cr.AIC = 440.5
AIC/N = 1.259

Table S4: Participant demographics stratified by randomized blocks 1 to 5 (N = 70)

**Characteristic**	**n (%)**				
	**Block 1**	**Block 2**	**Block 3**	**Block 4**	**Block 5**
**Age (years)**					
18 to 39	1 (7.7)	7 (50.0)	4 (28.6)	6 (42.9)	7 (46.7)
40 to 59	8 (61.5)	5 (35.7)	3 (21.4)	7 (50.0)	3 (20.0)
60 +	4 (30.8)	2 (14.3)	7 (50.0)	1 (7.1)	5 (33.3)
**Sex**					
Female	4 (30.8)	5 (35.7)	6 (42.9)	7 (50.0)	9 (60.0)
Male	9 (69.2)	9 (64.3)	8 (57.1)	7 (50.0)	6 (40.0)
**BMI kg/m^2**					
< 18.5 (underweight)	1 (7.7)	1 (7.1)	0 (0)	0 (0)	0 (0)
= 18.5 to 24.9 (healthy)	3 (23.1)	3 (21.4)	4 (28.6)	6 (42.9)	5 (33.3)
= 25.0 to 29.9 (overweight)	4 (30.8)	4 (28.6)	5 (35.7)	3 (21.4)	5 (33.3)
> 30 (obese)	5 (35.8)	6 (42.9)	5 (35.7)	5 (35.7)	5 (33.3)
**Current marital status**					
Single or partnered	5 (38.5)	5 (35.7)	5 (35.7)	6 (42.9)	7 (46.7)
Married or de facto	8 (61.5)	8 (57.1)	7 (50.0)	6 (42.9)	7 (46.7)
Separate, divorce, or widow	0 (0.0)	1 (7.1)	2 (14.3)	2 (14.3)	1 (6.7)
**Education**					
< year 12	1 (7.7)	3 (21.4)	5 (35.7)	4 (28.6)	3 (20.0)
HSC or equivalent to year 12	3 (23.1)	3 (21.4)	2 (14.3)	2 (14.3)	3 (20.0)
TAFE Diploma or Certificate	4 (30.8)	3 (21.4)	2 (14.3)	7 (50.0)	4 (26.7)
University Degree	5 (38.5)	5 (35.7)	5 (35.7)	1 (7.1)	5 (33.3)
**Current employment situation**					
Working full-time/part-time	5 (38.5)	4 (28.6)	5 (35.7)	7 (50.0)	5 (33.3)
Student	1 (7.7)	0 (0.0)	0 (0.0)	1 (7.1)	1 (6.7)
Retired (for age or medical reasons)	5 (38.5)	3 (21.4)	5 (35.7)	1 (7.1)	4 (26.7)
Not currently working (any reason)	2 (15.4)	7 (50.0)	4 (28.6)	5 (35.7)	5 (33.3)
**Ethic background**					
Aboriginal or Torres Strait Islander	1 (7.7)	1 (7.1)	0 (0.0)	0 (0.0)	0 (0.0)
Anglo-Caucasian	6 (46.2)	7 (50.0)	3 (21.4)	7 (50.0)	8 (53.3)
Asian	2 (15.4)	2 (14.3)	8 (57.1)	2 (14.3)	2 (13.3)
Middle Eastern	1 (7.7)	3 (21.4)	0 (0.0)	2 (14.3)	1 (6.7)
Pacific Islands	1 (7.7)	0 (0.0)	1 (7.1)	1 (7.1)	1 (6.7)
Other European	2 (15.4)	1 (7.1)	1 (7.1)	1 (7.1)	0 (0.0)
Other^	0 (0.0)	0 (0.0)	1 (7.1)	1 (7.1)	3 (20.0)
**Comorbidities**					
Diabetes	3 (23.1)	3 (21.4)	5 (35.7)	3 (21.4)	5 (33.3)
Cardiovascular disease	3 (23.1)	2 (14.3)	4 (28.6)	2 (14.3)	2 (13.3)
Hypertension	11 (84.6)	6 (42.9)	8 (57.1)	9 (64.3)	10 (66.7)
Stroke	1 (7.7)	0 (0.0)	1 (7.1)	1 (7.1)	1 (6.7)
Dyslipidaemia	3 (23.1)	2 (14.3)	6 (42.9)	1 (7.1)	4 (26.7)
**Gastro-related symptoms**	0 (0.0)	0 (0.0)	0 (0.0)	1 (7.1)	0 (0.0)
Coeliac disease					
GERD or reflux symptoms	5 (35.8)	8 (57.1)	7 (50.0)	6 (42.9)	8 (53.3)
Irritable bowel disease	1 (7.7)	2 (14.3)	2 (14.3)	2 (14.3)	3 (20.0)
Other *	4 (30.8)	5 (35.7)	5 (35.7)	4 (28.6)	5 (33.3)
**Smoking**					
Never smoked	9 (69.2)	7 (50.0)	9 (64.3)	10 (71.4)	12 (80.0)
Current smoker	0 (0.0)	1 (7.1)	0 (0.0)	0 (0.0)	0 (0.0)
Ex-smoker	4 (30.8)	6 (42.9)	5 (35.7)	4 (28.6)	3 (20.0)
**Time since last transplant (months)**					

**Transplant type**					
Kidney only	11 (84.6)	11 (78.6)	11 (78.6)	10 (71.4)	11 (73.3)
Simultaneous pancreas-kidney	2 (15.4)	3 (21.4)	3 (21.4)	4 (28.6)	4 (26.7)
**Donor type**					
Living donor	1 (7.7)	4 (28.6)	5 (35.7)	3 (21.4)	5 (33.3)
Deceased donor	12 (92.3)	10 (71.4)	9 (64.3)	11 (78.6)	10 (66.7)

BMI kg/m^2: body mass index kilograms per meter squared; **GERD:** gastroesophageal reflux disease; **HSC:** higher school certificate; **IQR:** inter-quartile range; **TAFE:** technical and further education; **<:** less than; **>:** greater than.

^ Other ethnicities: African American (1); Hispanic (1); Indian (1); Turkish (2).
*** Other GI symptoms:** bloating/distended stomach; bowel obstruction; chronic constipation; diarrhoea; feeling fullness; gastroparesis; gut pain; nausea; ulcerative colitis; urgency; vomiting.

Figure S1: Full electronic survey

i.

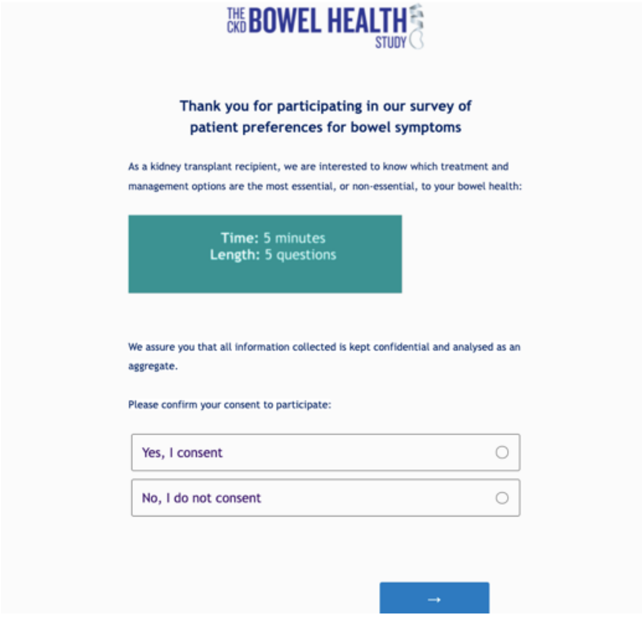

ii.

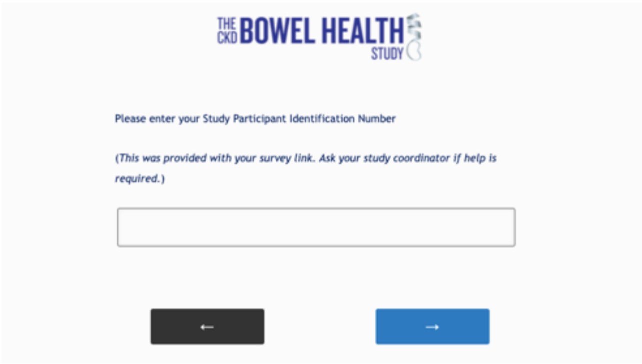

iii.

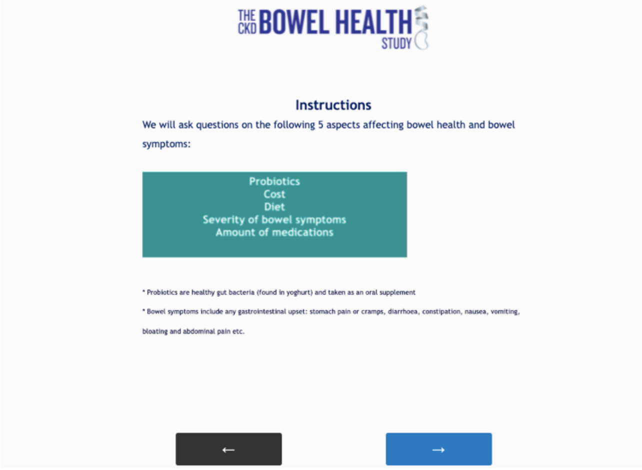

iv.

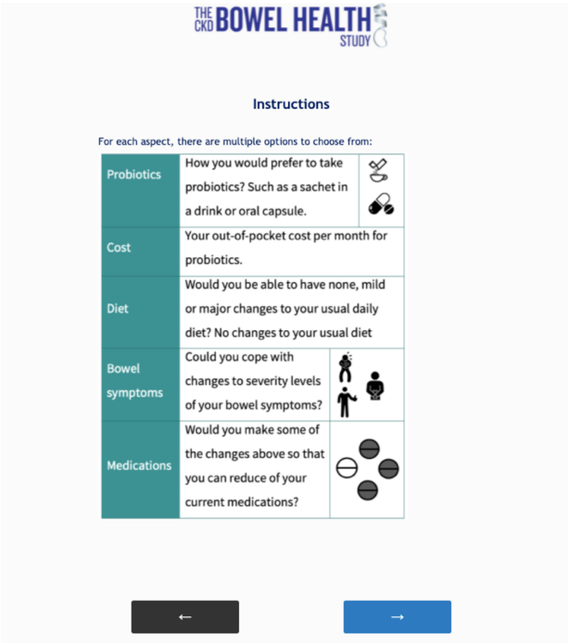

v.

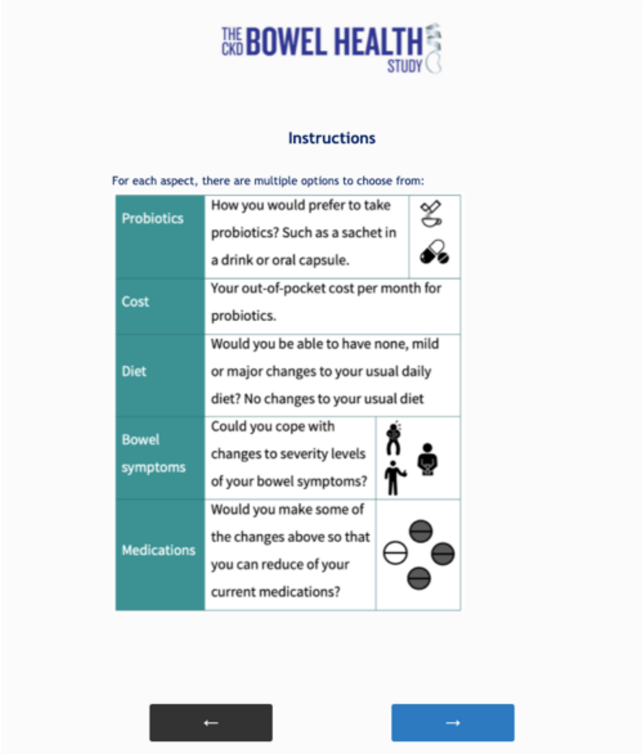
THE CKD BOWEL HEALTH STUDY
Instructions
For each aspect, there are multiple options to choose from:
Probiotics
How you would prefer to take probiotics? Such as a sachet in a drink or oral capsule.
Cost
Your out-of-pocket cost per month for probiotics.
Diet
Would you be able to have none, mild or major changes to your usual daily diet? No changes to your usual diet
Bowel symptoms
Could you cope with changes to severity levels of your bowel symptoms?
Medications
Would you make some of the changes above so that you can reduce of your current medications?
←
→

vi.

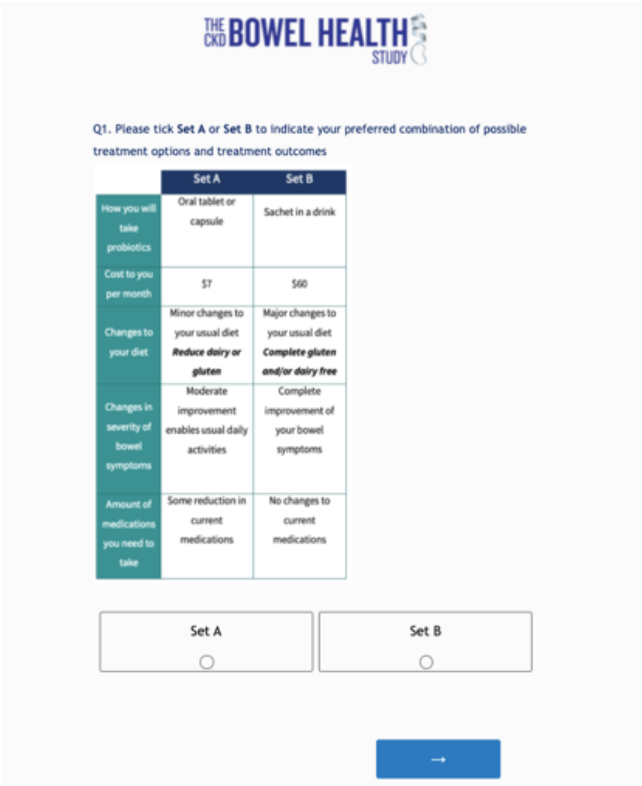
THE CKD BOWEL HEALTH STUDY
Q1. Please tick Set A or Set B to indicate your preferred combination of possible treatment options and treatment outcomes
Set A
Set B
How you will take probiotics
Oral tablet or capsule
Sachet in a drink
Cost to you per month
$7
$60
Changes to your diet
Minor changes to your usual diet Reduce dairy or gluten
Major changes to your usual diet Complete gluten and/or dairy free
Changes in severity of bowel symptoms
Moderate improvement enables usual daily activities
Complete improvement of your bowel symptoms
Amount of medications you need to take
Some reduction in current medications
No changes to current medications
Set A
Set B
→

vii.

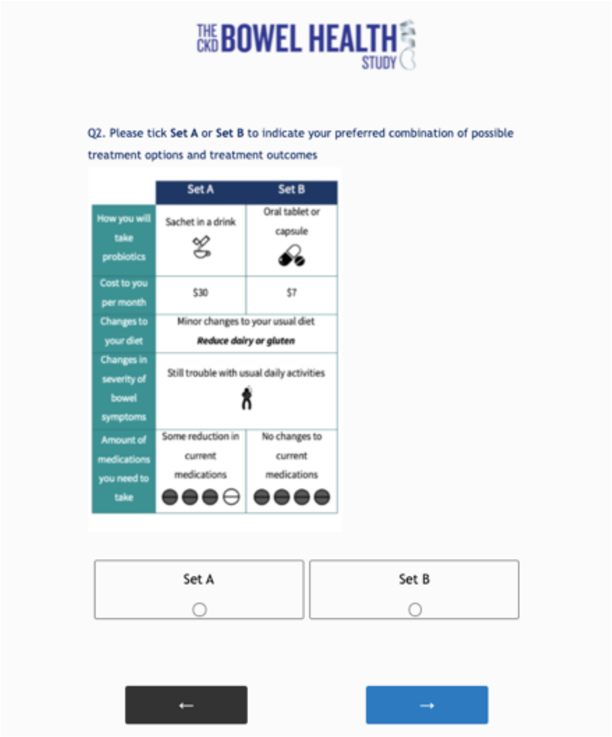
THE CKD BOWEL HEALTH STUDY
Q2. Please tick Set A or Set B to indicate your preferred combination of possible treatment options and treatment outcomes
Set A
Set B
How you will take probiotics
Sachet in a drink
Oral tablet or capsule
Cost to you per month
$30
$7
Changes to your diet
Minor changes to your usual diet Reduce dairy or gluten
Changes in severity of bowel symptoms
Still trouble with usual daily activities
Amount of medications you need to take
Some reduction in current medications
No changes to current medications
Set A
Set B
←
→

viii.

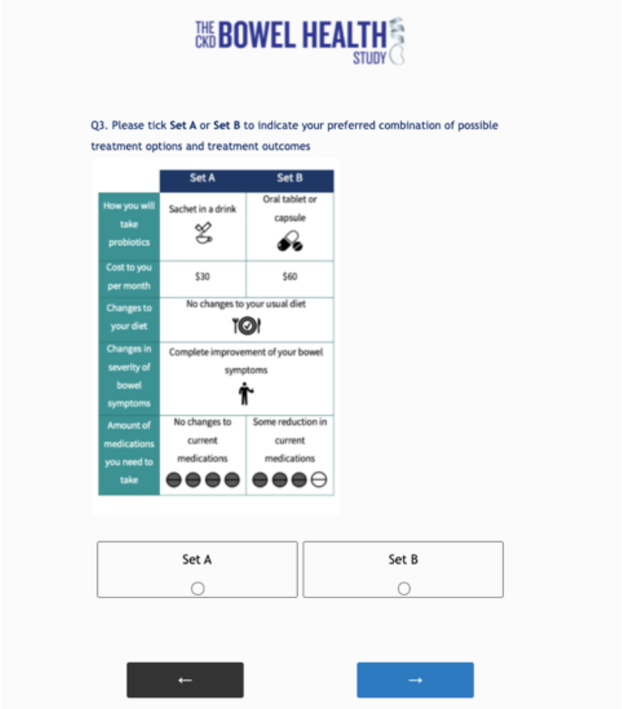
THE CKD BOWEL HEALTH STUDY
Q3. Please tick Set A or Set B to indicate your preferred combination of possible treatment options and treatment outcomes
Set A
Set B
How you will take probiotics
Sachet in a drink
Oral tablet or capsule
Cost to you per month
$30
$60
Changes to your diet
No changes to your usual diet
Changes in severity of bowel symptoms
Complete improvement of your bowel symptoms
Amount of medications you need to take
No changes to current medications
Some reduction in current medications
Set A
Set B
←
→

ix.

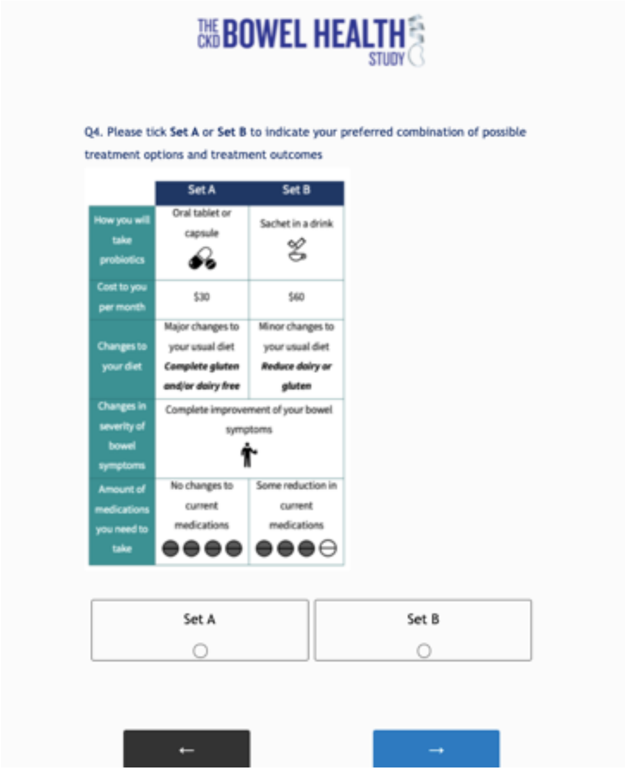
THE CKD BOWEL HEALTH STUDY
Q4. Please tick Set A or Set B to indicate your preferred combination of possible treatment options and treatment outcomes
Set A
Set B
How you will take probiotics
Oral tablet or capsule
Sachet in a drink
Cost to you per month
$30
$60
Changes to your diet
Major changes to your usual diet Complete gluten and/or dairy free
Minor changes to your usual diet Reduce dairy or gluten
Changes in severity of bowel symptoms
Complete improvement of your bowel symptoms
Amount of medications you need to take
No changes to current medications
Some reduction in current medications
Set A
Set B
←
→

xi.

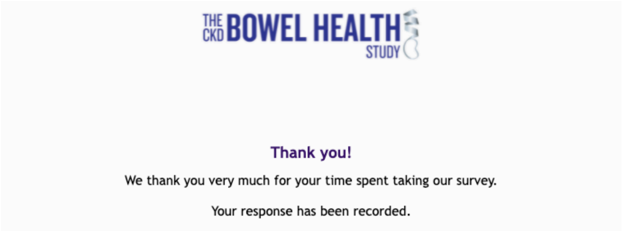
THE CKD BOWEL HEALTH STUDY
Thank you!
We thank you very much for your time spent taking our survey.
Your response has been recorded.

x.

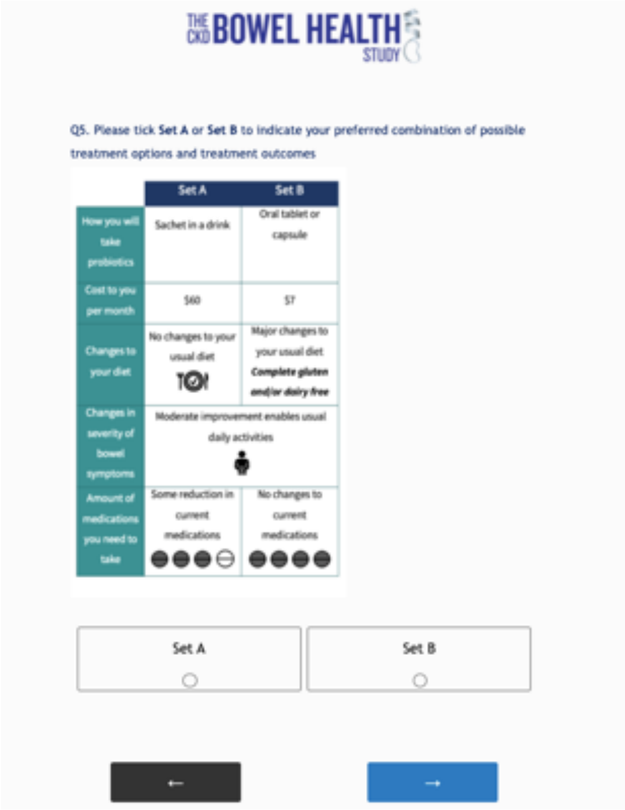
THE CKD BOWEL HEALTH STUDY
Q5. Please tick Set A or Set B to indicate your preferred combination of possible treatment options and treatment outcomes
Set A
Set B
How you will take probiotics
Sachet in a drink
Oral tablet or capsule
Cost to you per month
$60
$7
Changes to your diet
No changes to your usual diet
Major changes to your usual diet Complete gluten and/or dairy free
Changes in severity of bowel symptoms
Moderate improvement enables usual daily activities
Amount of medications you need to take
Some reduction in current medications
No changes to current medications
Set A
Set B
←
→

Figure S2: Study design and cohort flow chart

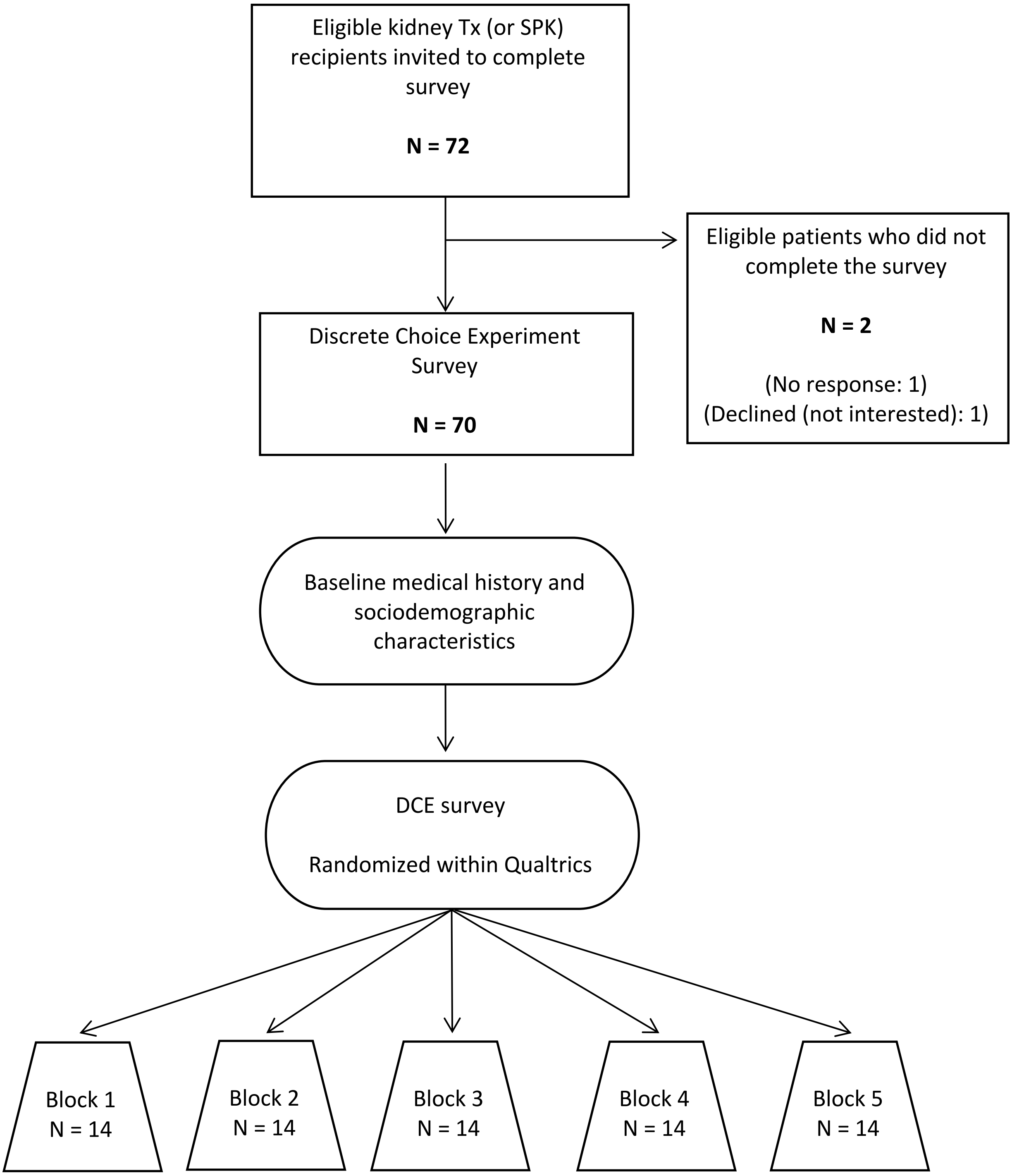

N: number of participants; **SPK:** simultaneous pancreas-kidney; **Tx:** transplant; **≥:** greater than or equal to.
